# Skeletal muscle as a pro- and anti-inflammatory tissue: insights from children to adults and ultrasound findings

**DOI:** 10.1007/s40477-024-00917-5

**Published:** 2024-06-21

**Authors:** Armando Di Ludovico, Saverio La Bella, Francesca Ciarelli, Francesco Chiarelli, Luciana Breda, Angelika Mohn

**Affiliations:** Department of Pediatrics, University of Chieti, Chieti, Italy

**Keywords:** Ultrasound, Myokines, Adipokines, Obesity, Inflammation

## Abstract

Previously regarded as a movement and posture control agent, the skeletal muscle is now recognized as an endocrine organ that may affect systemic inflammation and metabolic health. The discovery of myokines such as IL-6, released from skeletal muscle in response to physical exercise, is now one of the most recent insights. Myokines are the mediators of the balance between the pro-inflammatory and anti-inflammatory responses. This underscores the muscle function as a determinant of good health and prevention of diseases. Advances in ultrasound technology improved evaluation of muscle thickness, composition, and determining fat distribution. Combining imaging with molecular biology, researchers discovered the complicated interplay between muscle function, cytokine production and general health effects.The production of myokines with exercise showcasing the adaptability of muscles to high-stress conditions and contributing to metabolism and inflammation regulation. These findings have significant implications in order to provide improvement in metabolic and inflammatory diseases.

## Introduction

Scientific current research especially in the last decade revealed outstanding data that changed our perception about the skeletal muscles as a simple contractile tissue secretariat to the more complex endocrine organ [1]. This result springs from a growing knowledge that the human body may produce and release cytokines: interleukin-6 (IL-6) and some others in response to exercise [2]. This process play a critical function in maintaining the metabolic balance and immune reactions, placing the skeletal muscle in at the forefront of research into the exploration of homeostasis and the interactions between the immune system by mediating cytokine production and signal pathways [3].

The broader understanding of the topic derived from extensive studies is invaluable; the use of ultrasound contributed to the enhancement of that field and issues of muscle structure and function have now become much clearer [4]. Substantiated by the work of Hodges et al. the ultrasound imaging is considered as an essential tool for assessing the degree of muscle contractions, which was lacking in the traditional functions that were simple motion evaluations [5]. Furthermore, research validating the competencies of ultrasound in assessing body composition, as well as other parameters as muscle thickness, body fat distribution, and even structural changes, is an evident prove for the advanced of modern diagnostic technique [6]. Ultrasound and a molecular biology provide a better evaluation of the endocrine role of the muscle more especially in the context of ageing and chronic inflammation diseases. The association between the role of muscles, inflammation and chronic diseases is becoming more understood through studies that investigate muscle cytokines production, such as IL-6, and the implications of this process to chronic diseases [6]. For example, a study conducted by Pedersen and Febbraio, showed that muscle release IL-6 during exercise, with positive impact on inflammation and metabolism. Furthermore, Casey et al. showed that inflammation-associated skeletal muscle-derived cytokines participates in both systemic and local inflammation [7]. Such information showed how muscle cells are not just absorbing the immune system but are also interacting with it, implying an important significance. In conclusion, advancement in ultrasound and molecular biology have improved our understanding of the skeletal muscle, no longer considered with the only role of a organ of movement but also with a influencer of inflammation, metabolic balance, and overall health [8]. In this paper we explored literature from childhood to adulthood in order to obtain a full picture of how skeletal muscle’s and fat cytokines change over time in regulating metabolic and pro and anti-inflammatory balance. An improving in understanding this process, may provide an improvement in diagnostic and therapeutic assessment of health diseases related to an imbalance between muscle and fat cytokines production [9].

## Myokines

Skeletal muscle tissue showed a pro- an anti-inflammatory role, with implication from childhood to adulthood [10]. IL-6 is a principal myokine secreted from skeletal muscles during exercise and it is important for balancing inflammatory and anti-inflammatory reactions [11]. In addition, IL-6 increases insulin sensitivity and regulates the immune response [12]. Skeletal muscle is also another producer of other significant myokines that include IL-8, IL-15, and Fstl1 [1]. IL-8 is an angiogenesis promoting cytokine required for the formation of new vascular networks in muscle tissue and therefore muscle growth. One of IL-15 main functions is to stimulate muscle growth as well as fat metabolism [13, 14]. The function of Fstl1 is to promote tissue repair and regeneration [15]. Besides their specific functions, the muscle myokines together enhance lipolysis and endothelial function, vital for cardiovascular health [16].

**Fig. 1:**
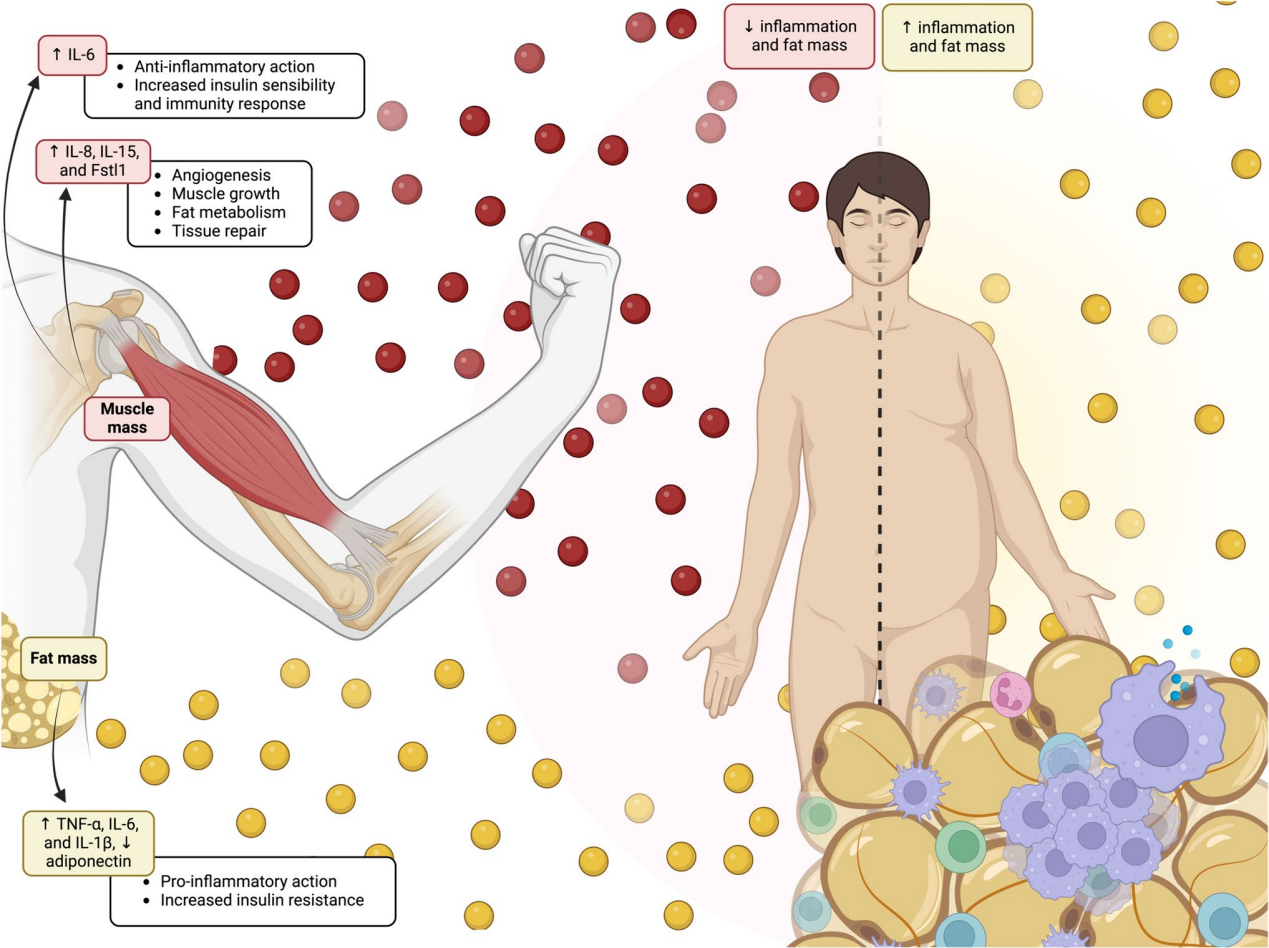
Impact of muscle and fat mass on human health. The muscle tissue and fat tissues differ in their response to inflammation. Elevated myokines levels such as IL-6, IL-8, IL-15, and Fstl1 promote muscle growth, angiogenesis, energy storage, and tissue cell repair. Fat mass accumulation cause adipokines release, leading to proinflammatory state and increased insulin resistance

The ultrasonography allowed monitoring of changes in muscle structure and function, thus revealing an exclusive view on the endocrine role of skeletal muscles [17]. Ultrasound gives a good assessment of muscle thickness and echostructure. Ultrasound also gives certain additional indirect data concerning muscles state and metabolic activities associated with secretion of myokines [18]. Ultrasound is a detailed muscle thickness and structural change quantifying tool, demonstrating how the skeletal muscle is a dynamic reactor to physical triggers with its endocrine role being underlined. The linkage of physical activity, myokine secretion, and systemic health responses shows that muscles are crucial in metabolism maintenance and overall systemic inflammation control [19]. Ultrasound studies findings, for example changes in muscle composition, and myokine secretion patterns, demonstrate not only exercise effect on the muscle but also its endocrine effect that is verifiable from childhood to adulthood (Fig. 1).

## Adipokines and myokines in obesity: impact on metabolic health

With the more advances of obesity research, the role of muscle and fat cytokines in systemic inflammation and the related development of metabolic diseases have become apparent [20]. With the more advances of obesity research, the role of muscle and fat cytokines in systemic inflammation and the related development of metabolic diseases have become apparent [21]. One of many adipokines, consistency levels of TNF-α, IL-6, and IL-1β, are usually increased among overweight individuals and they are related to insulin resistance, a process in which adipokines cause change in the way insulin is transmitted. However, the quoted cytokines, one, can inhibit insulin receptor substrates and, two, activate serine kinase pathways, that result in the reduction of insulin signaling [22]. Moreover, this increased production of pro-inflammatory cytokines leads to the transformation of macrophages into adipose tissue, where they take part in the inflammatory process [23]. Thus, a person who is overweight or obese is more likely to have a deficit in anti-inflammatory adipokines like adiponectin, and will, therefore, have insulin resistance in higher levels that are also inversely related to the amount of fat stored in the body [24]. While the reduction in adiponectin level is worrisome, due to its association with increased likelihood of metabolic syndrome, cardiovascular disease, and type 2 diabetes, the rest of the alterations are still to be studied [25]. On the other hand, muscle tissue not only serves as a metabolic organ, but also a secretmem exerted in physical activity is raised in muscle tissue it additionally acts as an endocrine organ similar to a pancreas, producing myokines during physical activity. Acting in an array of autocrines, paracrines, and endocrines have been demonstrated for these muscle-origin cytokines [1]. This group of myokines comprises IL-6, IL-15, and irisin proteins, which are secreted into the blood stream under muscle contractions. These proteins play an active role in the metabolic homeostasis. For example, IL-6 that is released by muscle during the exercise and has lipolytic and fatty acid oxidation effects [19]. An adipokine has its negative side when released by an adipose tissue that induces an inflammation, however in other situation body becomes anti-inflammatory when this adipokine is released by muscles during the mass [26]. IL-15 was linked to muscle growth and also the process of ‘browning’ of white fat where the white fat is converted into active brown fat through this mechanism. This is an adipose tissue type that is more efficient in calorie burning [27]. Irisin; a myokine, a cleaved membrane protein of FNDC5, has some proven to increase the energy expenditure and thus might act as an important anti-obesity and metabolic diseases factor [28].

The balance between the adipokines, the myokines intricately controls the general metabolism on the systemic level thus setting a complex between calorie burning, inflammation and metabolic-health rates. Conversely, the white adipose tissue produces the pro-inflammatory cytokines that cause toxinization of insulin receptor and result in higher insulin resistance in muscles [29]. This is an indication that adipokines and myokines are both equal in modifying the metabolism. Yet, myokines, a product of exercise secretion, might control these counter effects through enhancement of insulin response sensitivity and genesis of anti-inflammatory reactions [30].

These pathways implicate and characterize the intricate networks of cytokines that need to be determined in order to create potent management of obesity and its metabolic dysfunctions. Alternatives to therapeutic intervention in this regard may involve targeting cytokines to control their over-production or blocking the receptors to stop their damaging effect [31]. But the physical activity as the exercise has also other effects. It decreases the level of adipose tissue, which is associated with proinflammatory states. Further to this, physical activity enhances the positive effects of myokines. An example of this is that, one should keep their muscles and be fit, always as this is very important to overall health [16].

All things considered, the balance of the adipokine and the myokine is very important in the field of metabolic homeostasis, especially in the course of obesity diseases [32]. There are lots of factors that push this balance, which include the diet, activity, and the genetic predisposition, and their disturbance leads to systemic inflammation and insulin resistance, which are the key features of obesity. The data indicates that inequalities are inbuilt and have always remained in the course of social life [33].

## Muscle inflammation in multi-age populations

### Childhood

The growth period especially post muscle inflammation can become the life-defining period for body development and physiologic maturity [34]. Myokines, for instance IL-6, are in the lead of their function in co-ordination of balancing a pro-inflammatory response and an anti-inflammatory response within the muscle tissue [7]. The secondary molecules may be also very active in the mediation of the tissue stress adaptations to the physiological stresses like exercise, maintaining intrinsic repair and regeneration capacities, and finally triggering the process of renewal that follows traumatic injuries [35]. The signalling system of this complex is one of the most important features, enabling the organism to deliver a fast and controlled response to any type of damage and stress, in this way, the system will be trustworthy to protect muscle physiology from a variety of problems [36, 37]. Even though amino acids are the most essential, this phase becomes even more critical, because here myokines interact with hormones and growth factors, which are key drivers of muscle growth. They promote the proliferation of the satellite cell, their differentiation of the newly formed cells and their direct incorporation into the muscle fibers [38]. Stimulated by increased myokine levels, the muscle tissue releases growth factors that are involved in immediate repair and prepare the tissues for future stressers so instead of responding a once off repair they create an environment that supports long-term health [39].

### Adulthood

As individuals move into adulthood, the consequences of muscle inflammation evolve [36]. The modern life style in which ‘laziness’ rather than physical activity is typical is the total opposite of the physiological need of physical exertion which is one of the determining factors in the pro-inflammatory state of muscle tissue. Systematic physical activity supports healthy regulation of muscle secretion myokine muscle generators what is important for main ant-inflammatory conditions in the muscles tissue [16]. This adaptation is very crucial for the maintenance of metabolic health as it can help in increased insulin sensitivity, minimize the risk of chronic metabolic diseases, and improvement of body’s homeostasis [40]. When physical exercise is seen as an application that goes beyond the mere reduction of inflammatory markers to the induction of beneficial adaptations in the muscle fibers and an enhancement of the oxidative capacity, it becomes quite understandable why physical exercise improves energy utilization efficiency [41]. These adaptations result from the increased production of anti-inflammatory myokines and reduced metabolism of cytokines which are known for chronic inflammation and are dysregulated metabolically [42].

### Elderly population

Echo muscle thickness evaluation is a powerful tool that is used by the clinicians for diagnosis of sarcopenia in older patients. These assessments allow one to see structural changes in atrophic or inflamed muscles, offering an early reaction to defects that might cause muscle function to deteriorate, result in sarcopenia, and account for health-related diseases and disorders [43]. Despite of all, changes in skeletal muscle myokines secretion and inflammations over the life show that at all stages of our lives keel physiological system to normal [44, 45].

## Imaging muscle inflammation across the lifespan

Insight into ultrasonography amalgamation of a wide range of advanced imaging techniques beyond visualization, which aids in ecological assessment of inflammation due to obesity in skeletal muscles [46]. The application of ultrasonography at various life stages shows how it provides images of muscles and muscle cell function, which allows clinicians and researchers the safest and most effective way of observing the relationship between anatomical structure and pathological process [44].

The grayscale analysis, as a crucial element of ultrasonographic research, was specially created to investigate muscle echogenicity [47]. This technique includes the use of quantitative methods to determine the echogenicity (echogenicity is a feature that indicates how dehydrated and degenerated the muscle is, mostly caused by the fibrous tissues and the amount of fat deposit in the muscle), associated with the concentration of fibrous tissues and the amount of fat deposit in the muscle [48]. Such modifications demonstrate chronic inflammation; the latter is associated with echogenicity increasing, which is a sign of leaving normal muscle tissue behind [49]. Using ultrasonography, caliper is able to effectively determine changes in tissues as quantitative data allows to assess healthiness of the tissue and reveal early signs of histopathological alterations [17]. The road of life is marked with diverse challenges for the skeletal muscle from the increase in the demands as a child to the wearer out of the adulthood and to the physical test of maintaining integrity muscle with age. In particular, kids at an early age should be identified of inflammatory that comes out through ultrasonography and you should keep it as minimal as possible to prevent it from having a negative effect on growth and development [50]. The muscle ultrasound technique for evaluating the changes in muscle echogenicity and volume can provide an early detection of inflammatory myopathies that would allow the clinicians to intervene in the disease state and, as a result, an effective targeted treatment to alter the course of the disease [49].

While moving into adulthood the role of muscles changes from maturing to using them right and fighting inflammation [51]. There ultrasonography rules as a measure of effectiveness of muscle tissue rehab exercises. The underlying mechanisms of muscle-mediated anti-inflammatory effects can be demonstrated through ultrasonography in a defined image [52]. This measurement is apparent while the muscle is in motion as a result of physical activity. Ultrasound provides as a mirror reflecting the tissue's reactions to exercise, where the improvements in muscle architecture and a decline in the level of inflammatory markers are demonstrated and as a result it becomes obvious that physical activity preserves the organism against inflammation [52].

The golden years, as always, spawn their own issues, with sarcopenia and gradual muscle mass and function loss being the leading ones [53]. Ultrasonography using a feature that provides with muscle mass and quality assessment becomes an unreplaceable tool in detecting age-related pathology [54]. The ultrasonography method that includes the assessment of body areas, such as muscle thickness, area of cross-section, and intramuscular fat concentration, provides a comprehensive picture on the structural changes due to age and helps to define the influence of age on muscle alterations [55]. Elastosonography can as well analyze muscle stiffness that may appears during fibrosis [56].

In summary, from childhood to the elderly population, ultrasonography with the help of the utility of the technique provides a comprehensive depiction of the muscle health [57]. It goes beyond classic imaging features and provides a unique approach to observe how the inflammatory changes in the muscles due to obesity lasts throughout the life of an individual, from birth to old age [57]. Ultrasonography is not only a crucial part of detection, but also of the monitoring process, as well as the story of how active muscle fibers work with their environment and coping with chronic inflammation while growing.

## Ultrasound findings in obesity-induced muscle changes

The application of ultrasonography provides non-invasive information about skeletal muscle and adipose tissue throughout the lifespan (Table 1) [4]. These methods are based on the observation and classification of the progress of obesity-related changes in muscle and fat tissue in the body by utilizing a variety of scales and measurements. This suggests the interventions can be designed in a way which will aim at various stages of life (Table 2) [58].

**Table 1:** Muscular ultrasound changes in children and adult

Age group	Ultrasound findings	Implication	References
Children	Increased echogenicity	Indicative of inflammatory myopathies and suggests the presence of fibrous tissue and intramuscular fat, impacting muscle growth and metabolic health	[74, 75]
Children	Reduced muscle volume	May indicate muscle atrophy or diminished muscle development due to inflammation	[74, 75]
Adults	Increased echogenicity	Correlates with intermuscular adipose infiltration and fibrosis, linked to insulin resistance and decreased muscle function	[76, 77]
Adults	Changes in muscle architecture	Visualized alterations can indicate ongoing inflammatory processes, potentially affecting muscle strength and endurance	[76, 78]
Children and adults	Changes in muscles thickness and echo intensity	Indirectly reflects muscle health, metabolic activity, and the secretion of myokines within the muscle	[17, 18]

**Table 2:** Ultrasound metrics: assessment of muscle and fat tissue

Scale	Population	Description	Anatomical structures	Normal range	Variations from normal
Heckmatt Scale [60]	Children	Assesses muscle echogenicity from normal to increased fibrosis and fat infiltration	Quadriceps, especially the rectus femoris, due to its accessibility and size Medial gastrocnemius, for its propensity to show changes in echogenicity due to its composition and function	Grade 0 (normal) to Grade 1 (slight increase)	Higher grades indicate increased fat and connective tissue, suggesting muscle pathology
Muscle Quality Index (MQI) [79]	Adults	Combines measurements of muscle size with the degree of intramuscular fat for an overall assessment of muscle quality	Biceps brachii and triceps brachii in the upper extremities Rectus femoris and biceps femoris in the lower extremities, due to their size and role in locomotion	MQI < 25 (lower intramuscular fat)	Higher MQI scores may indicate sarcopenia or increased fat infiltration
Elastic modulus [77]	Adults	Quantifies muscle stiffness, often used in the assessment of fibrosis	Accessible and sizeable muscles such as the rectus femoris or biceps brachii These measurements are also useful in deeper muscles, but accessibility and the depth at which reliable measurements can be obtained may limit their use	< 8 kPa (indicative of normal tissue stiffness)	Increased values suggest greater tissue stiffness and potential fibrosis
Shear wave speed [77]	Adults	Measures the speed of shear waves through muscle, related to stiffness and used to assess fibrosis	Accessible and sizeable muscles such as the rectus femoris or biceps brachii These measurements are also useful in deeper muscles, but accessibility and the depth at which reliable measurements can be obtained may limit their use	1–3 m/s (indicative of normal tissue elasticity)	Higher values suggest increased muscle stiffness and potential pathology
Peak systolic velocity (PSV) [80]	All ages	Assesses the maximum blood flow velocity in muscle during the contraction phase of the heartbeat	Measurements are often taken in the major arteries supplying the muscle groups, such as the femoral artery for the lower limb muscles The brachial artery can be used for upper limb muscle assessments	Generally ranges between 30 to 100 cm/s, but this can vary widely based on the artery's size and the patient's hemodynamic status	Lower values may indicate reduced muscle perfusion and potential pathology
End-diastolic velocity (EDV) [80]	All ages	Measures the blood flow velocity in muscle during the relaxation phase of the heartbeat	Measurements are often taken in the major arteries supplying the muscle groups, such as the femoral artery for the lower limb muscles The brachial artery can be used for upper limb muscle assessments	Often ranges from 5 to 20 cm/s, again depending on the specific vessel and physiological conditions at the time of measurement	Lower values may indicate reduced muscle perfusion during relaxation
Resistive Index (RI) [62]	All Ages	Calculates the ratio of peak systolic velocity minus end-diastolic velocity to peak systolic velocity, indicating muscle vascular resistance	Similarly to PSV and EDV, RI measurements are typically taken in arteries within or supplying the muscle group of interest, such as the femoral artery for the lower limbs Small peripheral arteries within the muscle can also be assessed if the resolution of the ultrasound machine is sufficient	0.5–0.7 (indicative of normal vascular resistance)	Values outside the normal range suggest altered vascular resistance, which may reflect pathology

Contrast-enhanced ultrasound, a highly accurate very widely measuring technique, yields precise values of the cross-sectional area (CSA) and thickness of important muscles in the leg, like the rectus femoris or gastrocnemius [59]. These are the techniques to compare the measured results with the normal data in the children’s age group to identify the abnormalities. An example is the Heckmatt scale, it was originally designed to evaluate muscle quality in patients with specific neuromuscular diseases [60]. It provides an assessment of muscle quality evaluating echogenicity from normal group to severe group and higher score indicates more fat and connective tissues were infiltrated into the muscles [60].

Indeed, the absence of differences from well-regulated echo patterns and CSA measurements during this stage represents the loss of muscle strength due to obesity [61]. In this stage the Doppler ultrasound technology is used to assess the perfusion and microvascularity of muscle [62]. A Doppler ultrasound measures peak systolic velocity (PSV) and end-diastolic velocity (EDV) and these values are an indicator of the quality of the blood flow. Resistive indices, unlike measuring the flexibility of the vascular system or show rigidity [63].

For mid life adults, where obesity has a better chance to intensify its impact, Ultrasound is used as a tracker to measure changes over time. B-mode ultrasound is valuable and the temperature of muscle texture and internal fat can be seen with this [64]. In case of sonoelastography, which not only helps assembling shot qualitative and shot qualitative assessments of tissue stiffness but also reveals fibrosis [65]. Adopting standardized methods of assessment among parameters like modulus of elasticity or shear wave so as to give the density the material in numerical form makes the stiffness observed in the muscle tissue easier. The higher the value of fibrosis and inflammation, the better is the presence in the tissue for which it caters, therefore, the values provide crucial insights into the condition of the tissue [66]. Regarding obesity, or sarcopenia in aging, ultrasound test will delineate muscle cross-sectional area and thickness. This can be useful in sarcopenia and weight-related muscle changes diagnosis and treatment that help detect them at the early stages. Sarcopenia is a condition of the muscle loss related to age and weakening of muscles employed in weight-bearing activities. This assessment, in most cases, is based on the Muscle Quality Index (MQI) which considers muscle size as well as the degree and fat deposition in muscle [67]. MQI is the highly important parameter that not only estimates intramuscular fat status but also muscle area. Among the most important tasks of the study is the process of assessing intervention efficiency when it comes to successful prevention or treatment of sarcopenia [67].

In all the age groups the evaluation of subcutaneous and intrabdominal adiposity via ultrasound is very key understanding of adiposity distribution, which doctors normally refer to body’s underlying fat metabolism related disorders [64]. Measuring subcutaneous fat (SAT) thickness at the standardised anatomical sites, such as the abdomen or thigh, enables systemic SAT distribution assessment and provides an opportunity to reveal the association of metabolic risk factors with SAT accumulation [64, 68]. Sonoelastography does not only help in producing qualitative and quantitative parameters of tissue stiffness, but also detects fibrosis [65]. Adopting the standardized approaches for evaluation for such parameters as modulus of elasticity or shear wave to convert the density of the material into numerical form makes the stiffness observed in the muscle tissue more accessible. The higher the value of fibrosis and inflammation, the better is the presence in the tissue it is providing for, hence, the values are very important for the state of tissue [66]. Specifically, the prevalence of obesity, or sarcopenia in elderly, ultrasound test can define muscle cross-sectional area and thickness should be performed. The latter can be of great value in identifying diagnosis and treatment of sarcopenia and weight-related muscle changes that appear at the first stages. Sarcopenia is defined by age-associated loss of muscle mass and deterioration of muscles utilized in load-dependent activities. This assessment frequently includes the Muscle Quality Index (MQI), which considers muscle size and simultaneously the amount and deposition of in Muscle [67]. Metabolic Quality Index (MQI) is a crucial parameter that evaluates not only the condition of intramuscular fat but also muscle area. The process of assessing intervention efficiency in successful prevention or treatment of sarcopenia is one of the most important tasks of the study [67].

In all the age groups auditioning subcutaneous and intrabdominal adiposity via ultrasound provides very key understanding of adiposity distribution, which physician refer to as underlying body fat metabolism related diseases [64]. Measuring subcutaneous fat (SAT) thickness at the standardised anatomical sites, such as the abdomen or thigh, enables SAT distribution throughout the body estimation and provides an opportunity to reveal the association of metabolic risk factors with SAT accumulation [68].

Through the physician’s interpretation, validation of index and scale, physical examination that is then confirmed by ultrasonography, doctors can now identify, determine and measure the pathological changes in the body tissues due to muscle inflammation in obesity [69]. The analysis of different life stages allows us to comprehend the developing pattern as well as the effects of the obesity on skeletal muscles, and the clinicians attain the age-specific tailored approaches for personalised interventions in order to minimize the harm.

## Conclusion

Skeletal muscle is not a straightforward one but a rather complex one, which is constantly changing in terms of inflammatory response. It can synthesize cytokines which regulate many body functions and have effects on certain parts. These molecules, global inflammation mediators, include IL-6 that play an important role in muscle health and prevention of chronic ailments [70]. It is the endocrine function of muscle and physical activity that necessitates exercise as a role promoting muscle health and disease prevention through control of cytokine production [1]. Muscles can be seen clearly in ultrasound examinations while they are becoming inflamed. Such studies are the most known ways of monitoring inflammation of muscles. They could enable the medical staff to find out how to best aid their patients through their observation of the sound of the muscle cells contracting and that is associated with the inflammation [71].

Besides muscle mass assessment by ultrasonography methods, sarcopenia detection is important in evaluation of muscle health and inflammation related diseases [72].

The crosstalk between muscle mediated cytokines and the systemic inflammation is critical for it places the skeletal muscle as the main actor not only through movement but an active participant of metabolic well-being and of immunity responses regulation [42]. Systematic implementation of approaches designed for physiologic changes proposed for muscle in a way would make muscle tissue healthier, resulting in not only healthier metabolic profiles but also reduced chronic inflammation-associated diseases [73].

## Data Availability

Not applicable to this article as no datasets were generated or analyzed during the current study.
